# Genome-wide annotation of transcript boundaries using bacterial Rend-seq datasets

**DOI:** 10.1099/mgen.0.001239

**Published:** 2024-04-26

**Authors:** Andreas C. Lawaetz, Lauren A. Cowley, Emma L. Denham

**Affiliations:** 1 Life Sciences Department, University of Bath, Claverton Down, Bath, BA2 7AY, UK; 2 Milner Centre for Evolution, Life Sciences Department, University of Bath, Claverton Down, Bath, BA2 7AY, UK

**Keywords:** *Bacillus subtilis*, *Escherichia coli*, *Staphylococcus aureus*, Rend-seq, sRNA, pyRAP

## Abstract

Accurate annotation to single-nucleotide resolution of the transcribed regions in genomes is key to optimally analyse RNA-seq data, understand regulatory events and for the design of experiments. However, currently most genome annotations provided by GenBank generally lack information about untranslated regions. Additionally, information regarding genomic locations of non-coding RNAs, such as sRNAs, or anti-sense RNAs is frequently missing. To provide such information, diverse RNA-seq technologies, such as Rend-seq, have been developed and applied to many bacterial species. However, incorporating this vast amount of information into annotation files has been limited and is bioinformatically challenging, resulting in UTRs and other non-coding elements being overlooked or misrepresented. To overcome this problem, we present pyRAP (python Rend-seq Annotation Pipeline), a software package that analyses Rend-seq datasets to accurately resolve transcript boundaries genome-wide. We report the use of pyRAP to find novel transcripts, transcript isoforms, and RNase-dependent sRNA processing events. In *Bacillus subtilis* we uncovered 63 novel transcripts and provide genomic coordinates with single-nucleotide resolution for 2218 5′UTRs, 1864 3′UTRs and 161 non-coding RNAs. In *Escherichia coli,* we report 117 novel transcripts, 2429 5′UTRs, 1619 3′UTRs and 91 non-coding RNAs, and in *Staphylococcus aureus,* 16 novel transcripts, 664 5′UTRs, 696 3′UTRs, and 81 non-coding RNAs. Finally, we use pyRAP to produce updated annotation files for *B. subtilis 168*, *E. coli K-12 MG1655*, and *S. aureus 8325* for use in the wider microbial genomics research community.

**Impact Statement**

Non-coding RNAs encompassing *cis*- and *trans*-encoded segments are important regulators of gene expression and bacterial physiology. Many UTRs and sRNAs are missing or mis-represented in current annotation files, which greatly limits accurate quantification of transcript abundancies by RNA-seq and prevents optimal experimental design. pyRAP analyses Rend-seq data and enables rapid and accurate identification of start and stop sites genome-wide to single-nucleotide resolution. Improved sensitivity compared to available tools is achieved by employing parallel analysis of multiple datasets and assigning greater confidence to peaks present across multiple samples. Further sensitivity is achieved by incorporating coordinates obtained from other sources of transcriptome analysis for cross-validation. New annotation files updated to single-nucleotide resolution by pyRAP will provide a valuable tool to accelerate the study of RNA-binding proteins and non-coding RNAs and to identify ribonuclease processing sites.

## Data Summary

The pyRAP software to annotate Rend-seq datasets is freely available for download through the associated GitHub repository at https://github.com/ALawaetz/pyRAP. Updated annotation files created by pyRAP for *B. subtilis 168* (NC_000964.3), * E. coli K-12 MG1655* (NC_000913.2), and *S. aureus 8325* (NC_007795.1) are found in Tables S1–3. Supplementary tables and figures can be accessed through Figshare https://doi.org/10.6084/m9.figshare.25472437.v2 [1]*.*

## Introduction

Gene expression is a tightly regulated and dynamic process that allows bacteria to adapt to their surroundings by responding quickly to environmental stimuli. Physical and chemical signals such as cell density, temperature, DNA integrity, or nutrient availability are passed on through signalling cascades to sigma factors, specific transcription factors and regulatory non-coding RNAs to enhance or silence gene expression [2,3]. Historically, transcription factors have been studied more intensely than non-coding RNAs because proteins are easier to annotate due to their open reading frames [3,4]. However, to obtain a comprehensive understanding of the gene regulatory landscape of the cell, non-coding RNAs should not be overlooked. Regulatory RNAs play important roles in all aspects of cell biology and represent a multitude of species ranging from essential housekeeping genes such as 4.5 s RNA, RNase P, and tmRNAs, to *cis*-regulatory segments (UTRs of mRNAs), antisense RNAs, and *trans*-acting small RNAs (sRNAs) (reviewed in [3,5,11]).

Different bioinformatic tools are available that carry out automated annotation of genomes based on the DNA sequence, such as Prokka [12] or the NCBI prokaryotic Genome Annotation Process [4] that predicts protein coding genes, structural RNAs, tRNAs, sRNAs and other genomic features such as pseudogenes and mobile genetic elements. Prediction of non-coding RNAs relies on RFAM structures [13], a database of RNA families and consensus secondary structures, but such an approach inevitably overlooks non-conserved and non-structured RNAs [14]. Sequence-based predictive strategies are advantageous because they bypass laborious and expensive lab-based techniques. Expression studies, however, can be used to provide direct evidence of transcribed genes in a condition-dependent manner and studies using high-throughput techniques, such as microarrays [15,19] or RNA-seq [20,22] have identified hundreds of sRNAs.

Numerous studies in previous years have mapped transcription start sites (TSS) in both Gram-positive and Gram-negative bacteria with numbers in *E. coli* ranging from 16,539 TSS in one study [23] to 5197 and 1491 in other studies [24,25]. Similar discrepancies are observed in studies reporting TSS in *B. subtilis* with one Cappable-seq study reporting 5600 primary TSS in LB exponential phase condition [26] and another study using tiling arrays reporting 3242 transcriptional upshifts across 104 different growth conditions [15]. Differing numbers may in part be explained by condition dependence, varying sensitivities of different techniques, and batch effects [24,27]. However, with such wide-ranging numbers, the full extent of genome plasticity and the exact nature of transcription initiation, processing, and termination events in these bacterial species remains an open question.

RNA-seq has gradually become the method of choice for genome-wide quantification of gene expression and novel gene discovery because it is cheaper, more sensitive and provides better resolution than microarrays [28]. Various RNA sequencing methods have been designed specifically to map transcript boundaries to single-nucleotide resolution, such as the techniques dRNA-seq [29], Cappable-seq [26], Term-seq [30], and Rend-seq [31]. At present, neither GenBank annotation files nor species-specific genome browsers, such as the BsubCyc database [32], SubtiWiki [33], AureoWiki [34], or EcoCyc [35], are updated with the single-nucleotide resolution transcription data that is publicly available. This means that regulatory RNAs are either being overlooked or their start and stop sites are misrepresented. BSGatlas [36] is the most recent attempt to provide a unified *B. subtilis* genome browser, but still only provides single-nucleotide resolution for 21 % of listed transcription start sites and relies on tiling array data [15] (22 nucleotide resolution) for all remaining.

Imprecise annotations infer limitations to the detailed investigation of gene regulation and RNA processing events such as RNA turnover [37], production of sRNAs [38], and establishment of differential transcript levels within operons [39]. Numerous transcriptomic studies using both tiling arrays and RNA-seq have unravelled the targets of many RNases across multiple bacterial species by identification of differentially expressed genes [40,42]. Understanding RNA processing has advanced even further with methods such as RIP [43] and CLASH [44], which provide direct evidence of binding between RNA-binding proteins and their RNA targets. Finally, single-nucleotide resolution RNA-seq techniques have allowed identification of not only deregulated genes, but differentially expressed transcript termini [39].

**Fig. 1.**
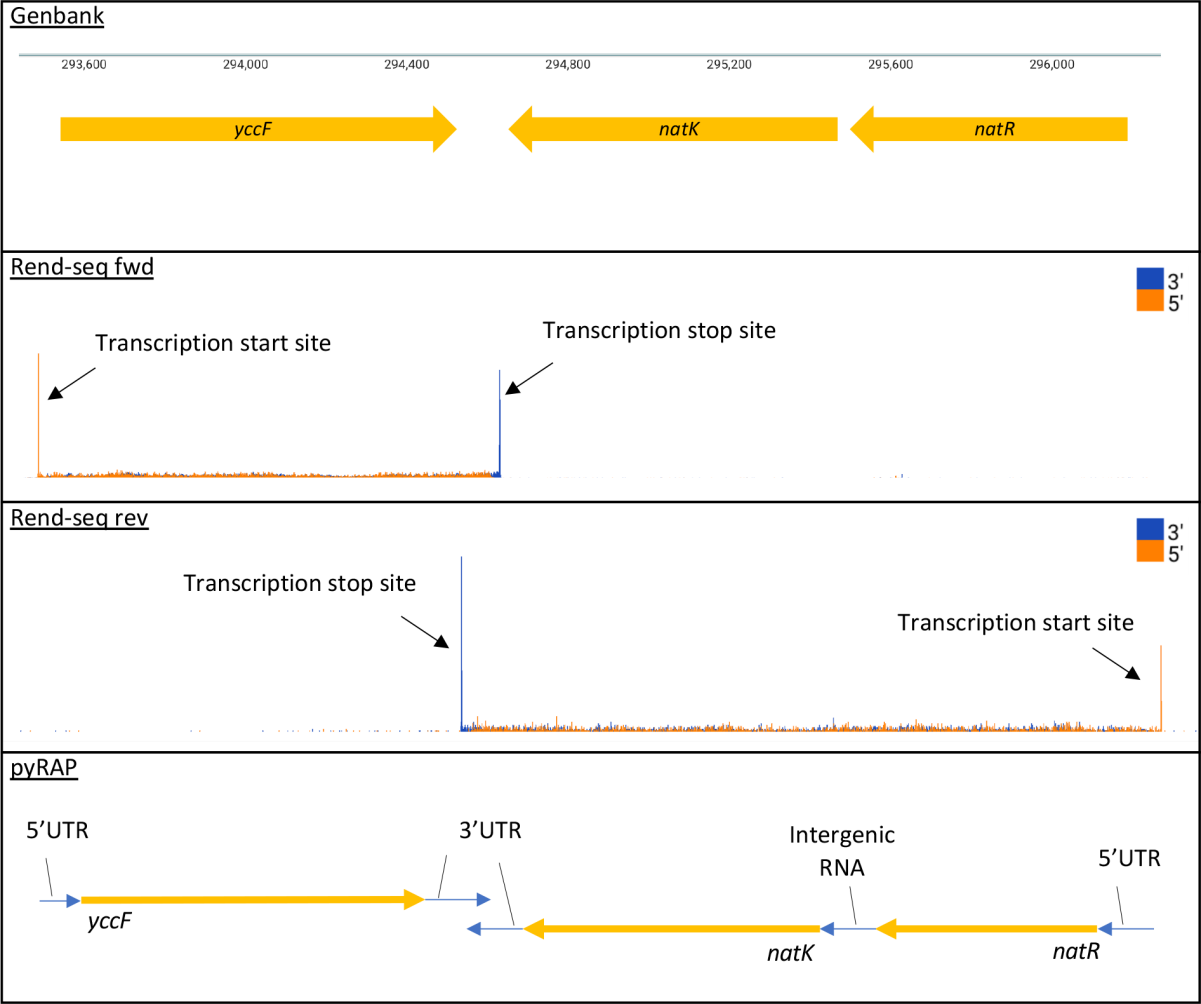
pyRAP annotates Rend-seq data genome-wide. Bacterial genes are often transcribed as multicistronic transcripts made up of coding sequences separated by intergenic regions of non-coding RNA. Coding transcripts are preceded by a 5′ untranslated region (5′UTR) and succeeded by a 3′ untranslated region (3′UTR). Rend-seq resolves transcripts’ start and stop sites through enrichment of 5′ reads (orange) and 3′ reads (blue) at transcript boundaries, respectively. pyRAP makes a genome-wide scan to call peaks at read values with *z*-scores above a defined threshold and draw annotations between them within operons.

Rend-seq is a method that simultaneously resolves 5′ and 3′ transcript boundaries in both primary and processed transcripts to single-nucleotide resolution. Rend-seq libraries are prepared by fragmentation of RNA, selection of short reads (15–45 nt), addition of 3′ linkers followed by reverse transcription, circularization, and amplification before finally performing short-read deep sequencing. Due to the random pattern of fragmentation, original transcript boundaries will be enriched in the final library relative to termini that are products of the preparative fragmentation step [31]. This method was initially developed to study the evolution of post-transcriptional regulation of protein synthesis rates [31]. A follow-up study [39] by the same group demonstrated that the post-transcriptional regulation necessary to produce differential protein levels within multicistronic operons are controlled by RNase Y with some genes being further dependent on the protein Y-complex for endoribonucleolytic cleavage. While some sRNAs and riboswitches were analysed in those studies, most of the regulatory RNA repertoire as reported by Nicolas *et al*. [15] has yet to be analysed. Non-coding RNAs constitute a quarter of all non-ribosomal reads in bacterial RNA-seq experiments [45], and while regulatory RNAs receive increasing amounts of attention, their functional characterization is still lagging behind their discovery [11]. Efforts to provide genomic coordinates of transcript start and stop sites for both coding and non-coding genes genome-wide and incorporate those into comprehensive updated annotation files have so far been limited. The original Rend-seq paper provides a pipeline to analyse operon architecture on a locus-by-locus basis [31]. It works by using a pileup script that takes sam files as input and counts the number of read termini mapping to the genome. Each read hence produces two values representing the genomic locations of its 5′ and 3′ ends. The total sum of this end counting is output to wig files that can be visualized using a customized genome browser (Fig. 1). A second script takes the wig files as input and creates a matlab figure showing operon architecture and relative isoform abundance in user-defined genomic regions. As the matlab pipeline has been designed to analyse differential gene expression within operons, it is not streamlined to output genome-wide annotation files. Another limitation of the software is that it relies on prior knowledge of operon coordinates, which complicates analysis of understudied genomic regions and annotation of novel transcripts. To address these issues, we present pyRAP (python Rend-seq Annotation Pipeline), an alternative and complementary high-throughput method for analysing Rend-seq data. pyRAP completely automates genome-wide mapping of transcript boundaries and novel transcript detection and outputs these in human readable annotation files that are compatible with many genome browsers and downstream analysis tools. Crucially, pyRAP provides increased sensitivity through cross-sample verification and incorporation of gene-coordinates obtained through different sources of expression data. The pyRAP software along with updated annotation files for *B. subtilis, E. coli* and *S. aureus* are freely available from GitHub, for wider use by the microbial genomics research community.

## Results

### Approach

We created pyRAP with the aim of genome-wide annotation and designed our pipeline to obtain maximum sensitivity while producing a minimum number of false positives. In contrast to the initial matlab script designed for Rend-seq data analysis [31], this is done by allowing incorporation of annotations from alternative sources (e.g. Cappable-seq, Term-seq, and tiling arrays) and through the use of differential threshold values for peaks unique to single samples, peaks occurring at the same position in multiple samples, and peaks evaluated in large and small windows. Peaks are called as transcript termini based on their z-score, the number of standard deviations the read value deviates from the mean in its downstream or upstream window (5′ and 3′ reads, respectively). Lalanne *et al*. used a default *z*-score of 12 calculated in 100 bp windows [31]. Using a large window to calculate *z*-scores, however, occasionally excludes the minor of two peaks positioned close to each other, because the larger peak drives up the mean (Fig. S5b, available in the online version of this article). With pyRAP, we therefore decided to use both 25 and 50 bp windows to calculate *z*-scores to avoid overinflation of the mean by neighbouring peaks. By comparing to the results reported by Lalanne *et al*. we found that termini producing *z*-scores above 13 in 100 bp windows, yielded *z*-scores down to 7.4 in 50 bp windows (Fig. S5a). Consequently, pyRAP is set to use a *z*-score threshold of 7 for start sites and 6 for stop sites. Lower *z*-scores are used for stop sites, because stop sites more often than start sites are confined to not just a single nucleotide but rather up to four nucleotides, which results in less end-enrichment [31]. To evaluate the performance of pyRAP on non-coding sRNAs, we examined the loci of known sRNAs, such as RosA, which has been experimentally shown to have multiple transcript isoforms [46]. We found that its longest isoform displays a small but clearly visible 3′ peak 39 bp downstream of a much larger peak (Fig. S5b). This smaller peak is only detected using a *z*-score cut-off of 5, calculated in a 25 bp window. To decrease the possibility of producing false positives, pyRAP flags all peaks with *z*-scores between 5 and 7. If multiple samples are analysed simultaneously and a flagged peak is present in more than one sample, the peak is called. Similarly, a peak can be verified using other sources of termini data, e.g. Cappable-seq and Term-seq. To further increase sensitivity, peaks can optionally be assigned to operons using externally provided operon coordinates, thereby ensuring that annotations are only made between termini belonging to the same operon. Using this feature, pyRAP lowers the read-density threshold from 0.25 reads/nt as used by the matlab script provided by Lalanne *et al*. [31] to 0.20 reads/nt. A complete overview of the pyRAP architecture and threshold values used is outlined in Fig. S1.

### Rend-seq coverage

Combining the datasets produced by Lalanne *et al*. [31] and DeLoughery *et al*. [39] we used pyRAP to create genome-wide annotation files with single-nucleotide resolution for *B. subtilis* in conditions of LB exponential phase, MSgg exponential phase and MSgg stationary phase, *E. coli* in MOPS complete medium exponential phase, and *S. aureus* in TSB exponential phase. Flagged peaks are verified using genomic coordinates of transcript start and stop sites from published studies using Cappable-seq [26], Term-seq [47,48], and RNA-seq data [24], and with operon coordinates created from tilling arrays [15,18].

Bacterial genes are organized into operons, the start and stop sites of which are resolved by Rend-seq. We calculated the percentage of the total transcriptome covered by Rend-seq data as the fraction of Rend-seq start and stop sites overlapping previously annotated operons out of the total. With that, we report Rend-seq coverage of 62 %, 43 %, and 45 % of the total transcriptomes of *B. subtilis, E. coli,* and *S. aureus*, respectively. pyRAP provides the option to output annotation files that are hybrids of annotations created by Rend-seq data and annotations created from other sources (e.g. tiling arrays) for genes without Rend-seq coverage. Comprehensive Rend-seq annotation files supplied from publicly available databases (SubtiWiki [33], EcoCyc [35], and AureoWiki [34]) and NCBI GenBank files for *B. subtilis* (NC_000964.3)*, E. coli* (NC_000913.2) and *S. aureus* (NC_007795.1) are provided in Tables S1–S3.

**Fig. 2.**
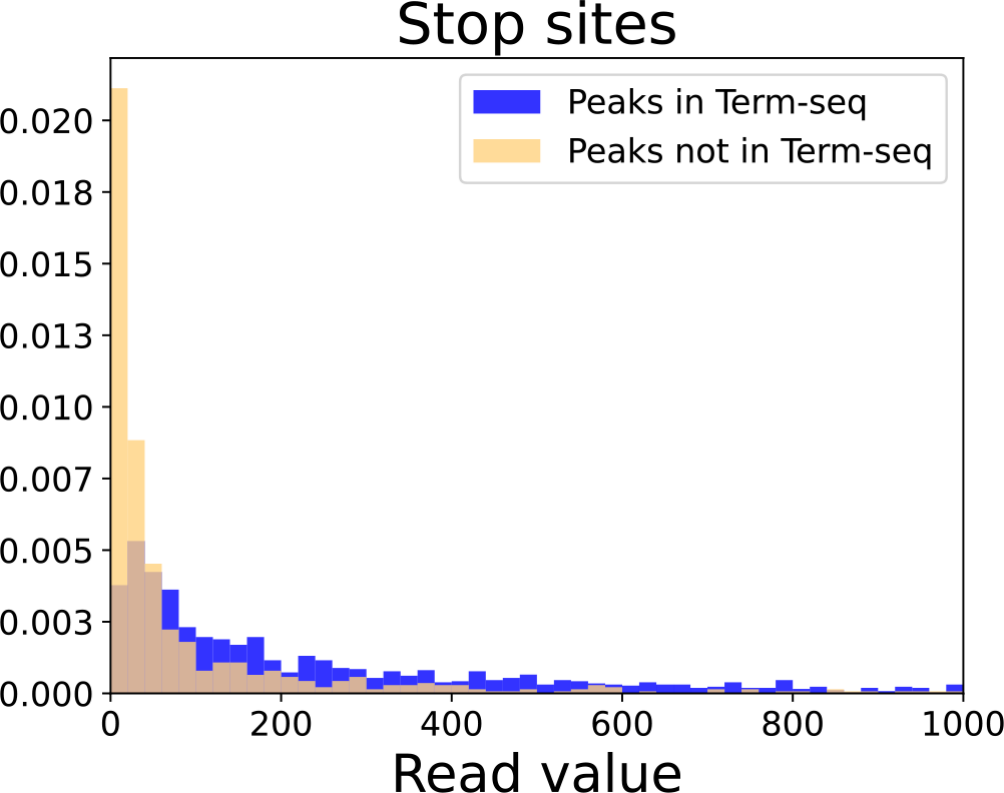
Rend-seq stop sites not detected by Term-seq have lower read values. Read value distributions of stop sites that are found (blue) or not found (orange) using Term-seq. Overlapping bars are shown in light brown.

With pyRAP, we report a total of 2944 start sites and 2352 stop sites in *B. subtilis,* 3116 start sites and 2041 stop sites in *E. coli,* and 962 start sites and 946 stop sites in *S. aureus* (Tables S1–S3). Running pyRAP with the *B. subtilis* LB condition only, we find 2654 start sites of which 145 had *z*-scores between 5 and 7 and were hence validated using the Cappable-seq data [26]. Similarly, of 2174 stop sites, 43 were flagged and validated using Term-seq [47]. To verify the total output of our pipeline and estimate the level of potential false negatives we compared overlaps with the complete Cappable-seq [26] and Term-seq [47] datasets. Unlike the Cappable-seq study, pyRAP only reports the highest peak within a 5 bp window. Hence, we report overlaps between methods if the reported coordinates are within 5 bp of each other. With that we find that 74 % of start sites in *B. subtilis* (LB exponential condition) resolved by Rend-seq are also reported in the Cappable-seq study by Warman *et al*. [26] and 50 % of the TSS reported with Cappable-seq in *B. subtilis* are also found in the Rend-seq dataset. With pyRAP, we can verify 64 % of stop sites in *B. subtilis* through comparison to stop sites reported in a Term-seq study [47] and 90 % of the 1549 stop sites from that study overlap with a Rend-seq stop site. Stop sites agreed between Rend-seq and Term-seq have generally higher read values than stop sites only found in the Rend-seq dataset (Fig. 2). Making the same comparison using the original Rend-seq publication [31], we found that only 76 % of the stop sites listed in the Term-seq study [47] overlap with the list of stop sites reported by Lalanne *et al*., which demonstrates that less false negatives is achieved by using pyRAP (90 % overlap).

### Annotation of UTRs

**Fig. 3.**
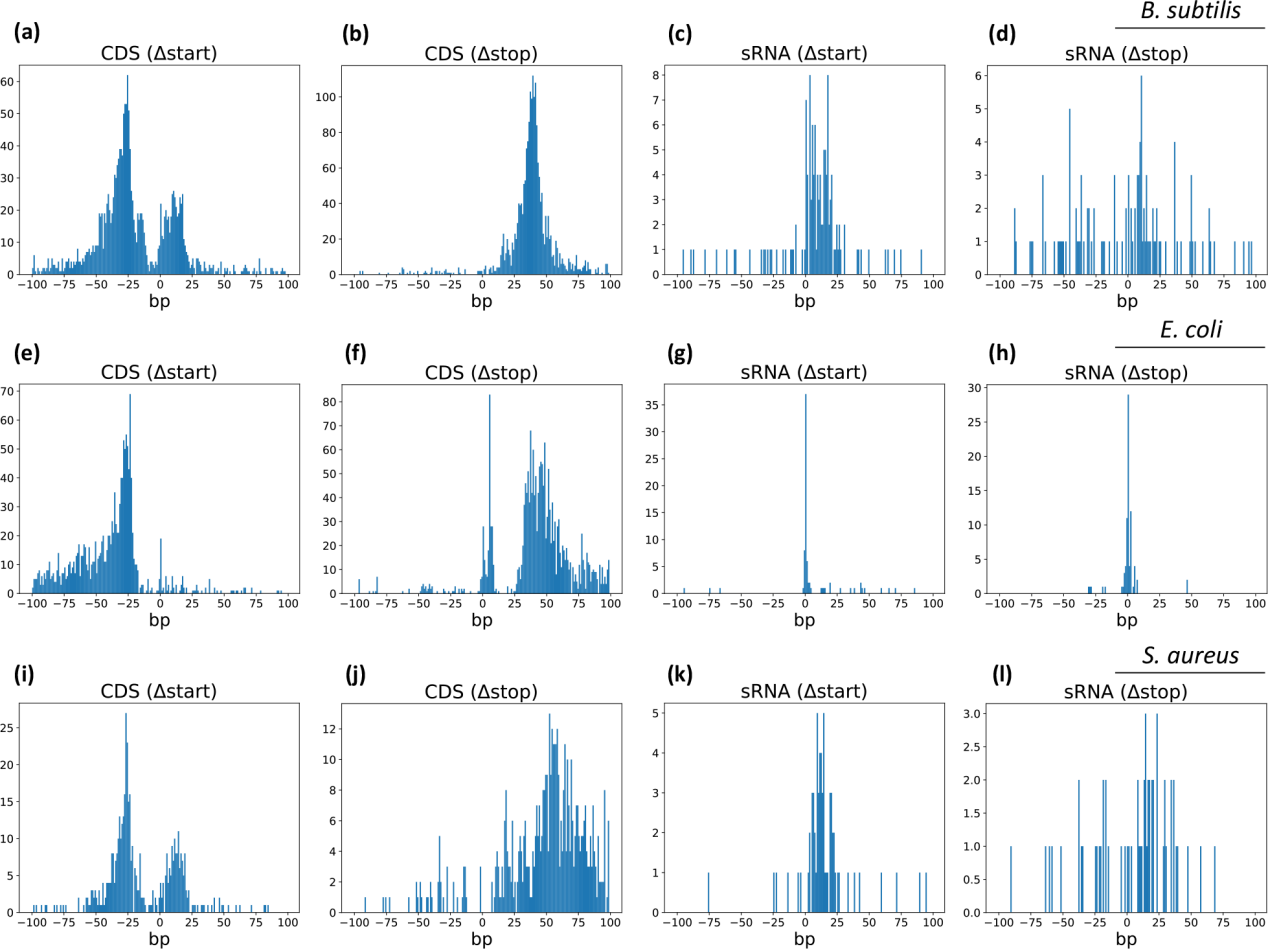
Numerous coding genes have currently no annotated UTRs. Distribution plots showing distance in coordinates between Rend-seq and current annotations [sources: SubtiWiki (*B. subtilis*), EcoCyc (*E. coli*), and AureoWiki (*S. aureus*) and NCBI GenBank] for CDS start sites (**a, e, i**), CDS stop sites (**b, f, j**), and sRNA start sites (**c, g, k**) and stop sites (**d, h, l**) in *B. subtilis* (top panel), *E. coli* (middle panel), and *S. aureus* (lower panel). Distance is calculated by subtracting current coordinates from pyRAP coordinates.

**Fig. 4.**
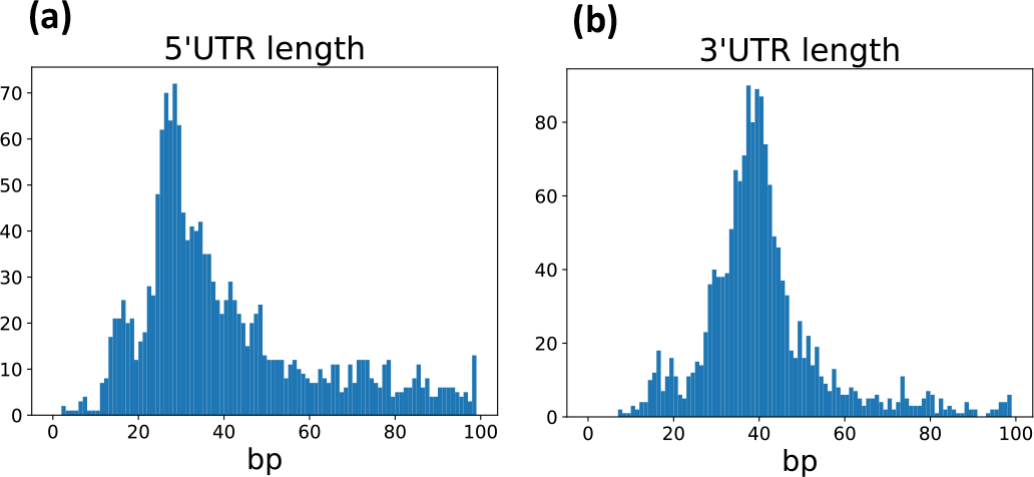
Distribution of 5′ and 3′UTR lengths. Distribution of lengths of 5′UTRs (**a**) and 3’UTRs (**b**) in *B. subtilis*.

**Fig. 5.**
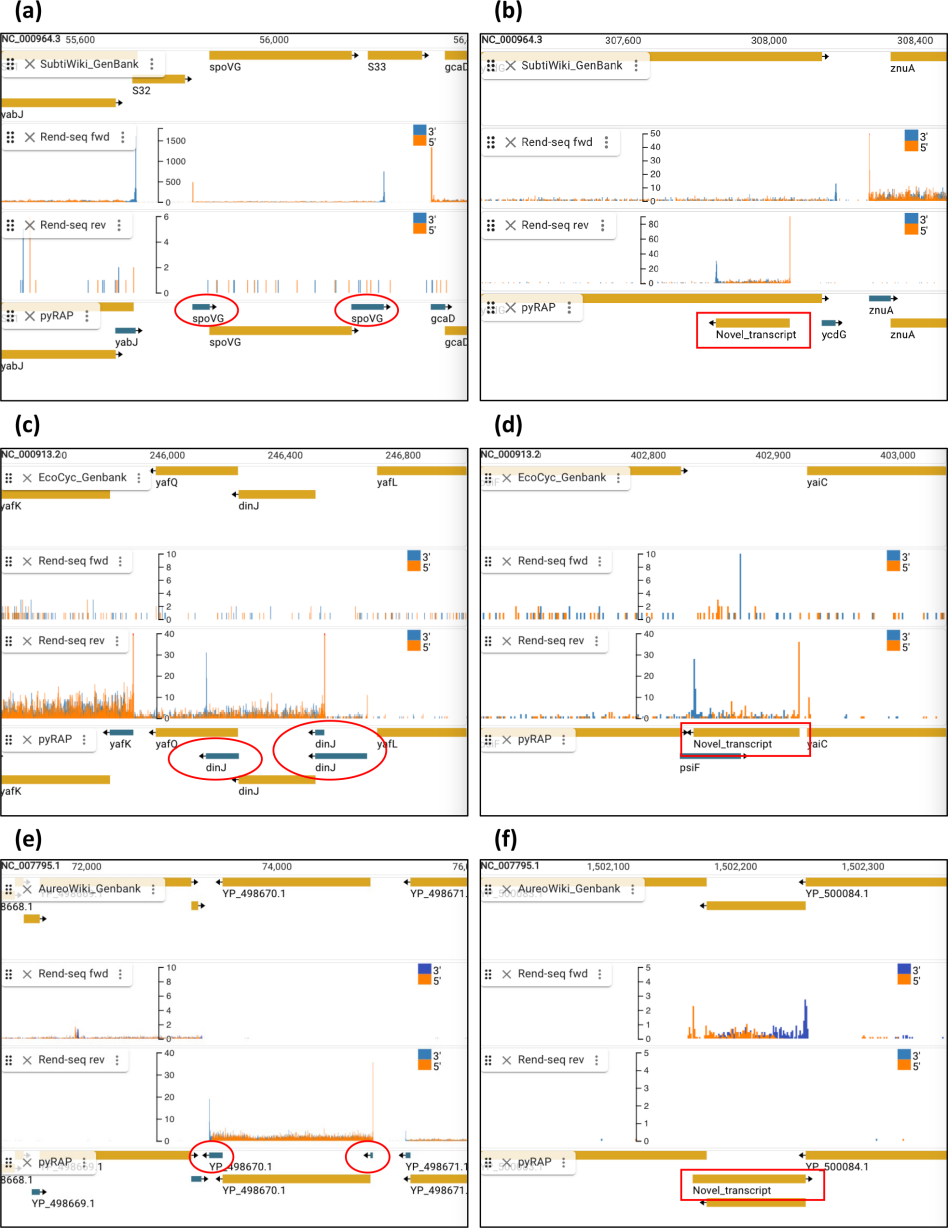
pyRAP annotates 5′ and 3′UTRs and detects novel transcripts. Jbrowse2 screenshots showing examples of 5′ and 3′UTRs (red circles) missing from current annotation files and novel transcripts (red squares) detected by pyRAP in *B. subtilis* (**a, b**), *E. coli* (**c, d**), and *S. aureus* (**e, f**). Top track in each screenshot shows current annotations, middle two tracks show Rend-seq wig files represented with XY coordinates (5’ reads: orange, 3’ reads: blue) for forward and reverse strand, and bottom track is the pyRAP annotation. Y-axis displays the pile up number of mapped end reads. The layout of figures is similar throughout unless otherwise described.

To identify unannotated UTRs, we calculated the distance from each Rend-seq annotation to the nearest GenBank or tiling array annotation (Fig. 3). For each species analysed (*B. subtilis, E. coli,* and *S. aureus),* a single annotation file was made for comparison by pooling the annotations that are available from GenBank with the annotations provided by SubtiWiki, EcoCyc, and AureoWiki*,* respectively, in order to encompass as many known annotations as possible. It is evident that numerous 5′UTRs are currently not annotated, as seen by a group of genes whose distance between their GenBank/tiling array start coordinate and Rend-seq coordinate is clustered around −25 nucleotides (GenBank/tiling array coordinate is downstream from Rend-seq coordinate) (Fig. 3a, e, i). A second clustering of datapoints in Fig. 3a, i, suggests that the genomic coordinates of UTRs that are currently annotated are on average 10 bp upstream from the position determined by Rend-seq. This general bias in the tiling array datasets is explained by the tiling step employed (22 nucleotides for *B. subtilis* and 18 nucleotides for *S. aureus*) and the fact that coordinates of up and downshift were defined to the 5′ end of hybridization probes [15,49]. Accordingly, the authors of those studies stated that up-shifts have a tendency to map 12 bp upstream of the actual transcription start site as determined by differential RNA seq [50]. Looking at stop sites (Fig. 3b, f, j) we see a clustering of genes at +40 bp, demonstrating a high number of coding genes without annotated 3′UTRs. This correlates with the pyRAP-determined size of 5′ and 3′UTRs in *B. subtilis* that are distributed around sizes of approximately 25 and 40 bp, respectively (Fig. 4). Similar correlations are seen for *E. coli* and *S. aureus* (Fig. S2). Using the customisable genome browser Jbrowse2 [51], we were able to visualize examples of 5′ and 3′UTRs that are missing from current annotation files, but are now resolved using pyRAP (Fig. 5, left panels). The non-coding annotations of the *B. subtilis* transcriptome is based on the Nicolas *et al*. tilling array study [15], which has a resolution of 22 nucleotides, and this is evident by the spread of the data seen for the start and stop sites of sRNAs (Fig. 3c, d). The *S. aureus* annotation is based on a tilling array study [34,49] with an 18 nucleotide resolution, and in accordance herewith we find for the majority of genes that the difference in coordinates between start and stop sites resolved by Rend-seq relative to those reported using the tilling array is centred around an 18 nucleotides relative difference (Fig. 3k, l). Interestingly, for *E. coli*, the majority of sRNAs that are already annotated have been annotated with single-nucleotide resolution precision (Fig. 3g, h).

### Novel annotations

**Fig. 6.**
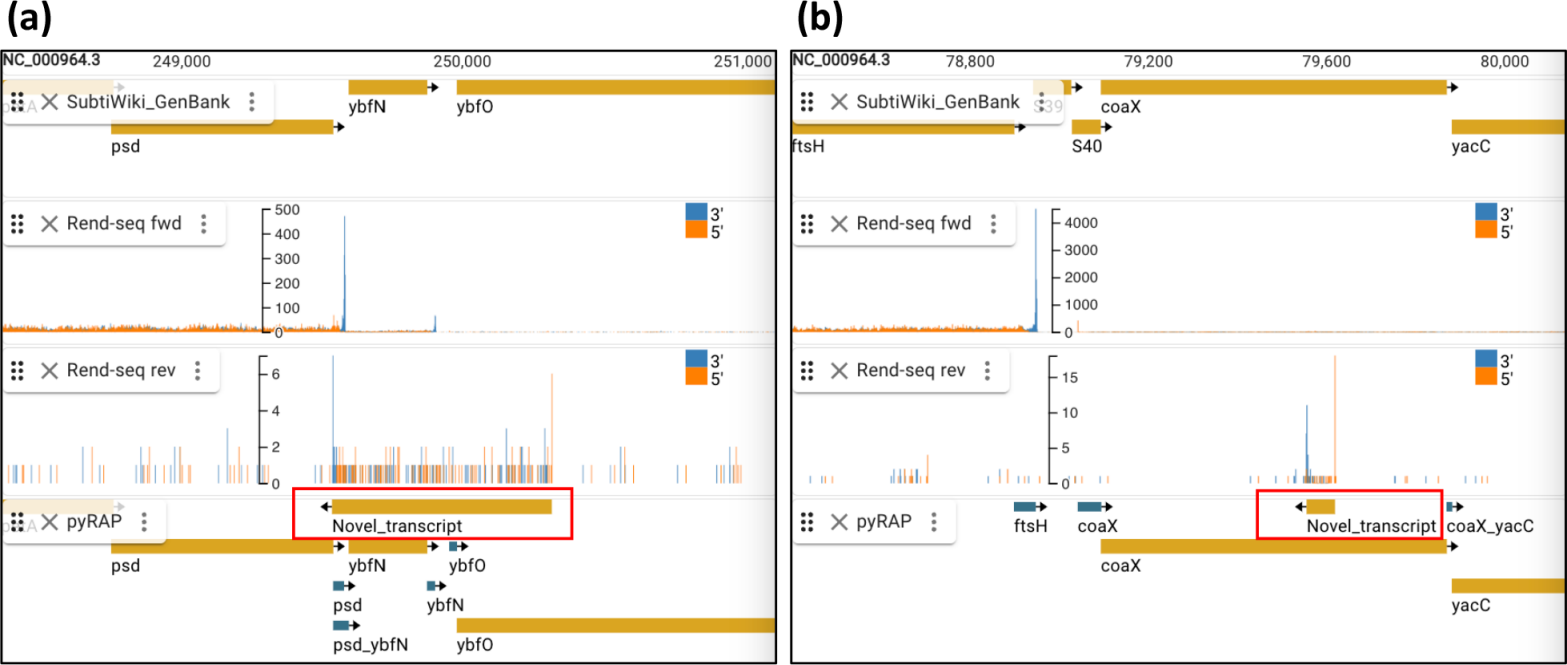
pyRAP detects low expressed genes. Antisense transcript to *ybfN* (**a**) and *coaX* (**b**), pyRAP detects novel start and stop sites, shown as representative examples.

The improved measures of pyRAP allows detection of start and stop sites in low expressed genes that were excluded in the analysis by Lalanne *et al*. [31]. In total, Lalanne *et al*. listed 1421 stop sites, of which 1387 (97.6 %) are also detected using pyRAP or are within two base pairs' distance (96.6 and 92.2 % overlap at 1 and 0 bp distance, respectively). Lalanne *et al*. further provided a list of 184 start sites that had been verified by primer extension experiments, of which 180 (97.8 %) are also found using pyRAP with 0 bp discrepancy. Hence, pyRAP fails to identify less than 3 % of start and stop sites identified using available software but, as shown here through comparison to stop sites reported using Term-seq, has an overall lower false negative rate (10 % for pyRAP versus 24 % for the matlab script [31]). Interestingly, despite the extensive work done previously on annotating the *B. subtilis* transcriptome across 104 conditions [15], pyRAP finds 63 transcripts that do not overlap with any genes annotated in the Nicolas *et al*. tiling array study and are therefore labelled here as novel (Fig. 5, right panel and Table S1). Of those 63 transcripts, 58 are expressed in the WT LB condition (the same sample as was analysed in the initial Rend-seq publication [31]) but only 23 of those transcripts were reported by Lalanne *et al*. [31]. Lowly expressed genes are overrepresented in this list of transcripts not reported by Lalanne *et al*. (examples shown in Fig. 6 and total transcript coverage shown in Fig. S4). Of the 40 novel transcripts not reported by Lalanne *et al*., 25 have transcript termini that can be verified using the Cappable-seq [26], the Term-seq [47], or the tilling array dataset [15] (transcriptional upshift). Of the remaining 15 transcripts without experimental evidence to verify their authenticity, 12 could be validated by identifying nearby predicted promoters or transcription terminator sequences (Table S4). In *E. coli*, pyRAP finds 117 novel transcripts that are not annotated in either GenBank or the EcoCyc database, and in *S. aureus* we report 16 novel transcripts that are not found in either GenBank or the AureoWiki database (Fig. 5, right panel and Tables S2 and S3).

To assess whether the relatively low number of novel transcripts detected in *S. aureus* is due to a lesser read depth, we calculated the total number of non-zero read values and divided by the genome size to determine the average number of reads per nucleotide (read density). *B. subtilis* WT in LB exponential phase was assayed both by Lalanne *et al*. [31] and DeLoughery *et al*. [39] and can hence be used for an approximate comparison of read depth across the two studies. In the study by Lalanne *et al*. [31] the average number of reads per nucleotide for *B. subtilis* was 0.31, whereas in the study by DeLoughery *et al*. [39] it was 0.18. For *E. coli* (Lalanne *et al*. [31]), read density was 0.26 reads/nt and in *S. aureus* (DeLoughery *et al*. [39]) it was 0.14 reads/nt. The varying read densities for *B. subtilis* illustrate differences in read depth across the two studies, which likely explains the high numbers of novel transcripts detected by pyRAP using the Lalanne dataset [31] compared to the DeLoughery dataset [39].

### sRNA processing

The tiling array study [15] reported 1583 putative regulatory RNAs in *B. subtilis,* which were categorized into segments mapping upstream and downstream of coding genes (5′ and 3′UTRs), between or within operonic genes (intergenic and intragenic segments, respectively) or outside coding genes (independent segments). The nature of tiling arrays makes it challenging to accurately resolve transcript isoforms and determine if a 5′ or 3′UTR may also exist in a form dissociated from the mRNA transcript from which it originates (5′ and 3′ derived sRNAs). In *B. subtilis* we have mapped the coordinates, with single-nucleotide resolution, of 2218 5′UTRs, 1864 3′UTRs, 161 non-coding RNAs, and 55 novel putative sRNAs (Table S1). Non-coding RNAs are labelled by pyRAP as sRNAs and do not include rRNAs, tRNAs or tmRNAs. These sRNAs encompass transcripts with a defined start and stop site that either map outside coding genes or overlap with mRNA transcripts without encompassing the full open reading frame of a previously annotated gene [3,5, 6]. Truncated transcripts that encode start codons but terminate before the stop codon are labelled as putative sRNAs though we recognize the possibility that they encode small peptides in alternative, yet unannotated open reading frames. In *E. coli* and *S. aureus* we have mapped the coordinates of 2429 and 664 5′UTRs, 1619 and 696 3′UTRs, 91 and 81 non-coding RNAs, and 60 and 33 putative sRNAs, respectively (Tables S2 and S3). pyRAP labels sRNAs according to their location relative to coding genes and whether they encode transcript isoforms. In summary, we find sRNAs derived from 5′UTRs (*B. subtilis*: *n*=56, *E. coli*: *n*=3, *S. aureus*: *n*=27), 3′UTRs (*B. subtilis*: *n*=15, *E. coli*: *n*=8, *S. aureus*: *n*=12), intergenic/intragenic segments (*B. subtilis*: *n*=10, *E. coli*: *n*=3, *S. aureus*: *n*=6), independent with isoforms (*B. subtilis*: *n*=37, *E. coli*: *n*=44, *S. aureus*: *n*=13), and independent without isoforms (*B. subtilis*: *n*=43, * E. coli*: *n*=33, *S. aureus*: *n*=23) (Tables S1–S3).

**Fig. 7.**
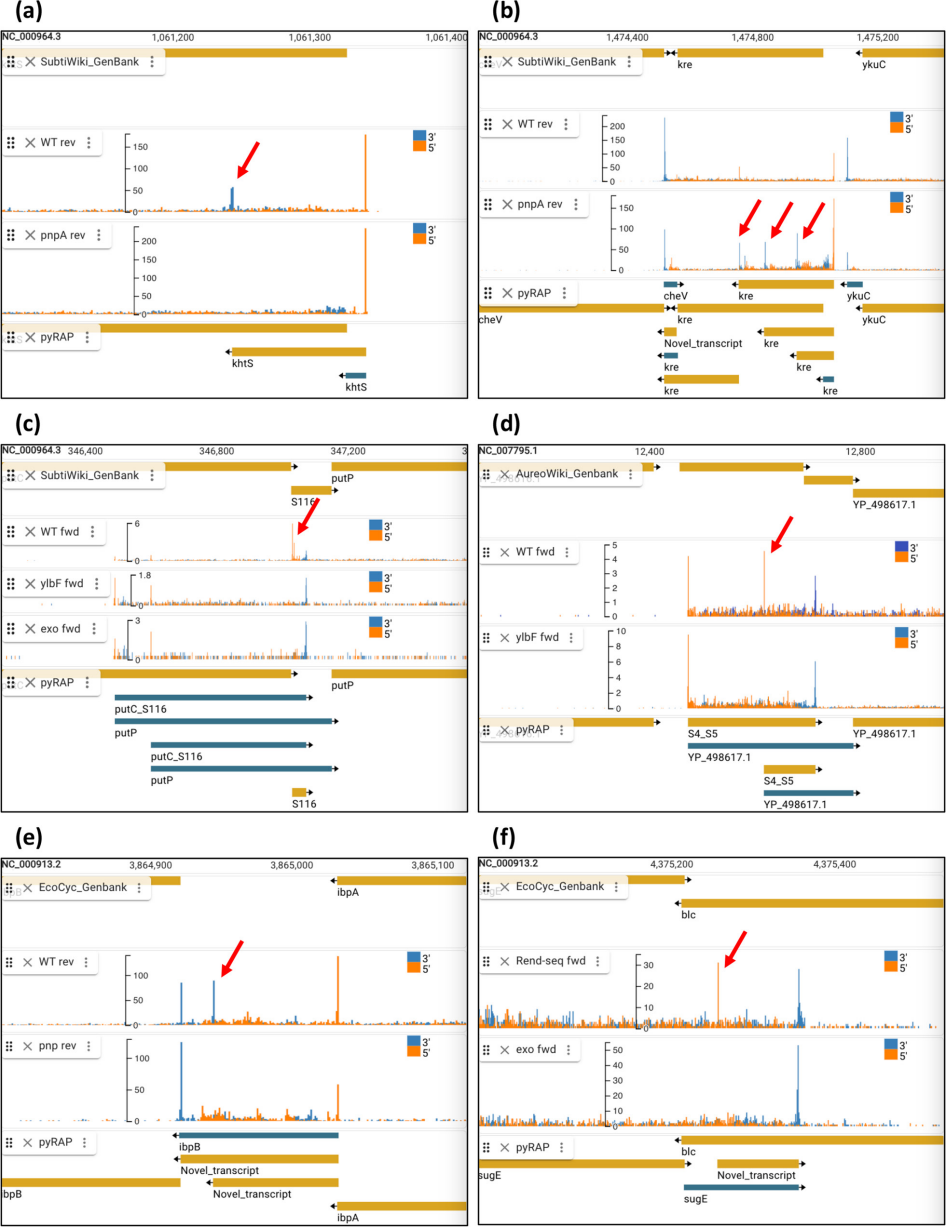
pyRAP identifies sRNA processing events. Processing (red arrows) of sRNAs and novel transcripts in *B. subtilis* [PnpA dependent (**a, b**), YlbF dependent and exo sensitive (**c**)], *S. aureus* (YlbF dependent (**d**), and *E. coli* [Pnp dependent (**e**) and exo-sensitive (**f**)]. In each screenshot, the same strand is shown in WT and mutant conditions (middle tracks).

To address the scope of RNase processing in the light of the total landscape of non-coding RNAs in *B. subtilis* including the 153 independent sRNAs reported in the tiling array study [15], we used pyRAP to output differentially expressed termini genome-wide in the published conditions of *ΔpnpA, Δrny, ΔylbF, ΔymcA, ΔyaaT,* and 5′ monophosphate-specific exonuclease treatment. The read density of Rend-seq datasets varies considerably between strains even within the same study (Fig. S3), which complicates the process of finding peaks unique to one sample that is not just a result of greater read depth. Therefore, to avoid making false-positive reports, we adopted a conservative approach and only included genomic regions with a minimum neighbouring read density of 0.20 read/nt in both WT and mutant strains. With that we found in *B. subtilis* a total of 17 sRNAs and putative sRNAs with differential expression patterns (Table S1 and examples in Fig. 7). Differentially expressed termini often correlate with differentially expressed genes [39], so to validate the putatively processed transcripts reported by pyRAP we compared these to published studies of deregulated genes in RNase mutants [40,41]. In total, we were able to verify, 15 of the 17 sRNAs reported as processed by pyRAP as having significantly deregulated transcript levels in RNase mutant strains (Table S1). The remaining two processed sRNAs are found within mRNAs that were reported by Liu *et al*. [40] with fold changes of 1.33 (*P*-value 0.11) and 1.83 (*P*-value 0.06) in a *ΔpnpA* strain.

**Fig. 8.**
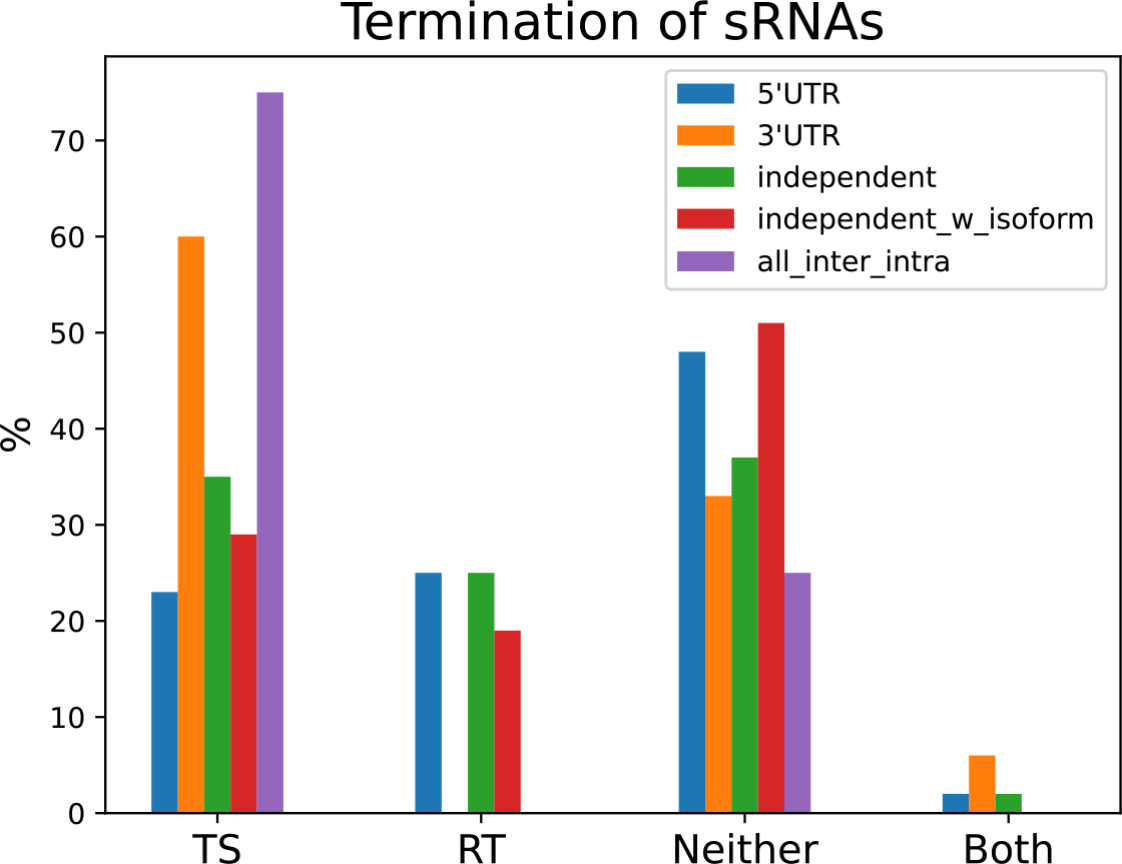
Independent sRNAs with transcript isoforms are more processed than independent sRNAs without isoforms. Percentage of sRNAs with predicted transcription terminators (TS), rho-dependent termination (RT), neither transcription terminator nor rho-dependent (Neither) or both (Both). Abbreviations: 5′UTR (5′UTR derived sRNA *n*=43), 3′UTR (3′UTR derived sRNA, *n*=15), independent (independent sRNAs without isoform, *n*=40), independent_w_isoform (independent sRNAs with isoforms, *n*=31), all_inter_intra (intergenic/intragenic sRNAs, *n*=8).

Searching the sequence from 60 bp upstream to 20 bp downstream of stop sites using the terminator finder ARNold [52], we find predicted transcription terminators for 34 % of all 161 sRNAs in *B. subtilis* (Table S1). For accurate comparison of transcription terminators versus rho-dependent termination, we limited the analysis to sRNAs expressed in the LB exponential condition (137 sRNAs total). We found that the mode of termination varies depending on sRNA type (Fig. 8). 60 % of sRNAs derived from 3′UTRs have a predicted transcription terminator at their stop site, whereas only 23 % of 5′ derived sRNAs have a predicted transcription terminator. Interestingly, 51 % of independent sRNAs with isoforms have neither a predicted transcription terminator nor display rho-dependent termination whereas for independent sRNAs without isoforms that number is 37 %. As expected, none of the 13 PnpA regulated sRNAs/putative sRNAs had intrinsic or rho-dependent termination signals (Table S1).

## Discussion

The perceived importance of non-coding RNAs in shaping the gene regulatory landscape of the cell has been increasing, especially with the emergence of high-throughput techniques, which greatly accelerated their discoveries. Regulatory RNAs often function through interactions involving the extreme ends of transcripts. Imprecise annotation of those regions may lead to important interactions being overlooked, prevent accurate functional characterisation, and misdirect research through incorrect *in silico* target predictions. We have presented pyRAP, a python written pipeline to annotate Rend-seq data genome-wide, and provided updated annotation files, which annotate 5′ and 3′UTRs, resolve transcript isoforms, detect novel transcripts, and identify processing events in *B. subtilis, E. coli,* and *S. aureus*.

Overall, we found that pyRAP annotated similar proportions of the transcriptomes in these different species of bacteria. A slightly higher coverage was achieved in *B. subtilis,* which had been assayed with Rend-seq across three different growth conditions, whereas *E. coli* and *S. aureus* experiments were done in one growth condition only. Further differences in coverage are explained in the varying densities of the individual libraries (Fig. S3). Incomplete coverage is expected as it has been shown that microbial transcriptomes are highly condition dependent [15].

The total number of start and stop sites in *B. subtilis* has been greatly debated, but comparing the number of termini reported across studies, needs to be carried out with caution, as different methodologies are designed with different aims. Different techniques of RNA-seq further varies in their method of library preparation, selection of reads, and final read depth. In the Cappable-seq [26] and Term-seq studies [47], a total read depth of 9 and 5 million reads per sample was used, respectively. In the study by Lalanne *et al*. [31], a much higher average total read depth per sample of 20 million reads was achieved, which, however, may partly be explained by the fragmentation step characteristic to the Rend-seq method. Still, Warman *et al*. [26] reported nearly twice as many start sites using Cappable-seq as we reported by analysing Rend-seq, which is unexpected as the method of Cappable-seq detects primary transcripts only, whereas Rend-seq also resolves processed sites. Discrepancies in numbers may at least partly be explained by the fact, that the Warman *et al*. study [26] reported individual start sites separated by as little as two nucleotides whereas pyRAP only reports the highest peak in any five nucleotide neighbourhood. Our report of 2944 start sites for three conditions is similar, though on the high end, when compared to the tiling array study by Nicolas *et al*. [15], who reported 3242 upshifts across 104 conditions. The greater sensitivity and higher resolution of RNA-seq compared to tiling arrays may at least partly explain the apparent discrepancy between these figures. Less controversy revolves around the number of stop sites. Nicolas *et al*. reported 2126 downshifts [15], and with Term-seq, 1549 termination sites have been listed with single-nucleotide resolution [47]. In LB exponential phase (the same condition as for the Term-seq study), pyRAP reports 2176 stop sites. This discrepancy is possibly explained by a difference in read depth as we find that Rend-seq stop sites overlapping with Term-seq have higher expression values than stop sites only found in the Rend-seq dataset (Fig. 2).

Improved sensitivity of pyRAP compared to the original matlab script is demonstrated by a greater overlap with Term-seq (90 % versus 76 %). At the same time, less than 3 % of start and stop sites detected using the original matlab script [31] fail detection by pyRAP. Increased sensitivity potentially comes at a cost of decreased specificity, if only achieved by lowering cut-off values. pyRAP, on the other hand, gains its sensitivity through cross-sample and cross-method verification and by calculating *z*-scores in smaller windows, thereby allowing detection of closely positioned termini. Considering the increased sensitivity of this pipeline compared to the matlab script [31], and keeping in mind that the number of start and stop sites provided in this study lands on the middle of the scale of what has been reported in other studies, suggests that pyRAP has a low false-positive rate.

With pyRAP we have increased the number of regulatory non-coding segments in *B. subtilis* from 1583 as reported by Nicolas *et al*. [15] to 4298 including 5′UTRs, 3′UTRs, sRNAs and putative sRNAs. The true number of regulatory RNAs may be even bigger as pyRAP annotates transcripts that partially overlap open reading frames in the 3′ end of genes as CDS. Such transcripts, however, may possibly be transacting sRNAs or dual transcripts, having sRNA activity while also encoding small peptides [53]. It should also be noted that experimental evidence is needed for the verification of transcripts annotated as intragenic and intergenic sRNAs by pyRAP, as such annotations might not represent actual transcripts, but rather be misconceived as such from the overlapping of two larger transcripts.

For the majority of sRNAs annotated using pyRAP, we did not find differentially expressed termini in RNase mutants or exo treated samples. It is, however, possible that the true level of processing is underestimated by pyRAP due to the challenge of direct comparison between samples of varying read depth (Fig. S3). Durand *et al*. [41] found 471 non-coding segments with transcript levels significantly deregulated by more than twofold in either RNase III, RNase J1, or RNase Y mutant strains. In total, 46 of the sRNAs reported by pyRAP are found on this list of deregulated non-coding genes, but it is challenging to conclude merely from transcript levels, whether the effects are direct or indirect. Liu *et al*. [40] outlined the regulon of PnpA, however, only examined coding genes, leaving it an open question the role of PnpA in regulating independent sRNAs. As pyRAP found only 17 sRNAs with differentially expressed termini, of which seven were found in a *pnpA* mutant and, hence, may be just decay intermediates, it appears that the majority of sRNAs are not processed by RNase Y or PnpA. It is expected, though, that many are indeed processed, as we found 59 sRNAs without intrinsic or rho-dependent termination signals. Further to suggest processing is that many of these sRNAs have sequence overlap with mRNAs or are isoforms of independent sRNAs. Therefore, it may be that processing of sRNAs in *B. subtilis* is mainly regulated by yet uncharacterized RNA-binding proteins. A number of proteins have in recent years been described as being RNA binding in *B. subtilis* and other Gram-positive bacteria*,* namely SpoVG [54], Kre [55], Jag [56], and KhpA [56], and as their functions remain largely unexplored it will be interesting to see what role they play, if any, in sRNA processing.

Non-coding RNAs are hotspots of gene regulation and precise, comprehensive annotations provide greater foundations for precise read mapping in RNA-seq experiments. This includes experiments employing RIP-seq techniques such as CLASH [57] and CRAC [58] that are particularly sensitive to accurate annotations, as they rely on very short reads that are often found in non-coding regions. Part of analysing CLASH and CRAC data involves identifying putative RNA:RNA interactions, which is done *in silico*, but naturally, the output of such algorithms is only as good as the input. As many UTRs are not annotated in GenBank, many bioinformatic pipelines employ automated assignment of UTRs at fixed lengths. Alternatively, RNA-seq data is used to estimate the boundaries, however, this is not accurate because read depths at 5′ and 3′ ends of transcript are generally lower compared to coding sequences. As regulatory RNAs function through secondary structures that are highly sequence dependent and as UTR lengths vary considerably (as shown in this study), we expect that great improvements can be achieved in sRNA target predictions using single nucleotide resolution annotation files. Knowing the exact position of transcript start and stop sites will be of great value in the design of many experiments and for characterising UTR function.

We add pyRAP to the transcriptomics toolbox, offering rapid, accurate and genome-wide analysis of Rend-seq data employing commonly used output formats compatible with many genome browsers and downstream applications. pyRAP provides improvements to current software by offering increased sensitivity and easy user customisation. Furthermore, unlike available software, pyRAP does not require input coordinates, thereby simplifying novel transcript detection and analysis of formerly understudied genomic regions. For the benefit of the microbial genomics community and the general advancement of RNA-research, the pyRAP software is open access and freely available from GitHub along with easy user instructions and updated annotation files for *B. subtilis, E. coli* and *S. aureus*.

## Methods

### pyRAP architecture and setup

The pyRAP package comprises a number of scripts used in unison within the script ‘pipeline.py’. A flowchart of the pipeline is outlined in Fig. S1. Customization of threshold values, modules to include, and output formats are achieved using the script ‘EXAMPLE_Terminal_input.py’. The entire package can be downloaded from GitHub at https://github.com/ALawaetz/pyRAP.git along with instructions on how to instal pyRAP and view wig files in JBrowse.

### Peak calling

Wig files are analysed in the ‘Peak_calling.py’ script, which calculates a *z*-score at each genomic position based on its 25 and 50 bp downstream or upstream read values, for 5′ and 3′ reads, respectively (Fig. S5). Start and stop sites are called at positions with *z*-scores above the threshold values set with the options --*z*_start, --*z*_stop, and --*z*_small. Default *z*-scores in 50 bp windows are seven for start sites and six for stop sites. In 25 bp windows, *z*-score five is default for both starts and stops. If multiple starts or multiple stops are called within the same +/−5 bp window, only the highest peak is kept. Peaks with *z*-scores below *z*_ii (default=7) are flagged and needs further verification before being called.

### Verification of flagged peaks

Verification is done with the module ‘options_unique.py’, which removes flagged peaks that are found in *N* number of samples only, where *N* is set by the user with the option --unique. Other means of verification include comparison to published transcriptomic data and is done with the modules ‘SupplyCapTerm.py’ and ‘Draw_GFF3_supply_w_capterm.py’. Using the options --compare_starts, --compare_stops, --operon_starts, and –operon_stops, annotations from multiple sources can be used simultaneously, e.g. Cappable-seq, Term-seq, and tiling arrays. Using the search_distance flags, the user is able to set the stringency of the comparison by telling pyRAP the number of bp discrepancy that still passes for a match. By default, using the search_distance flag verifies peaks that are within +/−5 bp of a coordinate in the alternative annotation file and the search_distance_0 flag verifies peaks that are within 30 bp.

### False discovery rate

To reduce the potential number of false positives, pyRAP includes a module ‘fdr.py’ that excludes peaks with read values that might be expected solely by chance. Read values expected by chance are calculated by taking the sum of all read values surrounding a peak in a 50 bp window and distributing the reads at random across a hypothetical 50 bp gene. Based on the random distribution, a threshold value *T* is calculated as the sum of the mean read value and standard deviation multiplied by *z* (*T*=mean(read values) + *z* * standard_deviation(read values). The number *z* is set by the user with the option --fdr (default=2). Peaks with read values below the threshold T are discarded.

### Assigning peaks to operons

Operon coordinates can be provided to pyRAP by the user or generated automatically by pyRAP. Automated annotation of operons by pyRAP is done in the ‘Make_RendSeq_operon_annotation.py’ module. Operons are made on the basis of read density between peaks in the sample defined using the --guide flag. Read density between peaks is calculated as the fraction of non-zero values to twice the distance between the peaks (as each genomic coordinate has two read values; a 5′ and a 3′ read). Operons begin where the read density between a start and stop site is above the threshold set by the user with the --operon_cutoff flag (default is 0.8) and they end where the read density between a stop site and its downstream peak is below the threshold. Peaks are assigned to the operons with which they overlap. Peaks that are not assigned to operons by this method are assigned to the nearest operon (downstream for start sites and upstream for stop sites). An exception to this rule is unassigned start sites where there is an unassigned stop site downstream or an unassigned stop site with an unassigned start site upstream in which case these ‘orphan’ peaks are believed to represent the boundaries of the same low expressed transcript. Annotations are made between orphan peaks if the annotation does not span an annotated operon.

### Transfer of features

Names, features, and attributes can be given to pyRAP annotations by providing an alternative annotation file (e.g. a GenBank file) with the flag –old_annotation flag in the ‘post_processing.py’ module. Annotations that completely overlap with one or multiple coding genes are assigned the feature CDS and are subsequently subcategorised into CDS and UTRs based on the CDS coordinates in the alternative annotation file. Annotations not assigned as CDS are assigned the feature of the annotation of which it overlaps in the alternative annotation file. Transcripts without any overlap are labelled as novel transcripts. Transcripts that overlap with multiple different features in the alternative annotation file are labelled ‘transcript’. Some transcripts are expressed in a way so that a start site can be detected but where a clearly distinguished stop site cannot, e.g. transcripts regulated by rho-dependent termination. In such cases and if an alternative annotation file is provided, a ‘hybrid’ annotation is made connecting the Rend-seq resolved start site to the nearest stop site of any overlapping annotations. To make it clear which coordinates are resolved with single-nucleotide resolution, hybrid annotations are marked in the ‘phase’ column of the pyRAP output with the value 0 and a note is made in the attributes stating what coordinate has single-nucleotide resolution. A similar approach is used for transcripts where only the stop site coordinate has been pyRAP-resolved.

### pyRAP output

The output of pyRAP is annotation files in GFF3 (default) or GTF file format. For each sample, two files are created, one showing all possible transcript configurations within operons based on connecting every start and stop site, and another file where CDS transcripts have been sub-categorized into UTRs and coding sequences. Another two files are created; the first file is named ‘All_conditions’, which include annotations from all the samples analysed and the second file with the extension ‘supplied_with_AA’ where annotations of genes without Rend-seq coverage is supplied with an alternative annotation file (AA), e.g. GenBank. Using the ‘--output All’ option it is possible to examine all the files made by pyRAP including the *z*-scores used to call start and stop sites.

### Accession codes and annotation files

The following genome assemblies and accession codes were used: *B. subtilis 168* (NC_000964.3), *E. coli K-12 MG1655* (NC_000913.2), *S. aureus 8325* (NC_007795.1). Wig files used to verify pyRAP and create new updated annotation files were from the study by Lalanne *et al*. [31] and DeLoughery *et al*. [39], which can be found in the NCBI Gene Expression Omnibus with series numbers GSE95211 and GSE108295, respectively. Annotation files used for comparison and for transfer of names and features to the pyRAP annotation were obtained from SubtiWiki [33], EcoCyc [35], AureoWiki [34] and GenBank. Annotations from EcoCyc are in *E. coli K12 MG1655 version three* format and were converted to *version two* format using the lift-over tool available on the EcoCyc platform. Updated pyRAP annotation files are found in Tables S1–3 and on the associated GitHub page (https://github.com/ALawaetz/pyRAP.git). Default pyRAP parameters were used in all instances, except for *S. aureus* where --*z*_ii=6 and --search_distance_0=50 were used. For all species, --unique=1 was applied.

## Abbreviations

CDS: Coding Sequence
CLASH: Cross-linking Ligation and Sequencing of Hybrids
CRAC: Cross-linking and analysis of cDNAs
pyRAP: Python Rend-seq Annotation Pipeline
RIP-seq: RNA Immunoprecipitation and Sequencing
sRNA: small non-coding RNA
TSS: Transcriptional Start Site
TTS: Transcription termination site
UTR: Untranslated Region
